# Exercise ameliorating myocardial injury in type 2 diabetic rats by inhibiting excessive mitochondrial fission involving increased irisin expression and AMP‐activated protein kinase phosphorylation

**DOI:** 10.1111/1753-0407.13475

**Published:** 2023-09-18

**Authors:** Bin Wang, Chen Zhao, Yuanxin Wang, Xin Tian, Junjie Lin, Baishu Zhu, Yalan Zhou, Xin Zhang, Nan Li, Yu Sun, Haocheng Xu, Renqing Zhao

**Affiliations:** ^1^ College of Physical Education Yangzhou University Yangzhou China

**Keywords:** exercise, irisin, mitochondrial fission, myocardial injury, type 2 diabetes

## Abstract

**Purpose:**

Though exercise generates beneficial effects on diabetes‐associated cardiac damage, the underlying mechanism is largely unclear. Therefore, we prescribed a program of 8‐week treadmill training for type 2 diabetes mellitus (T2DM) rats and determined the role of irisin signaling, via interacting with AMP‐activated protein kinase (AMPK), in mediating the effects of exercise on myocardial injuries and mitochondrial fission.

**Methods:**

Forty 8‐week‐old male Wistar rats were randomly divided into groups of control (Con), diabetes mellitus (DM), diabetes plus exercise (Ex), and diabetes plus exercise and Cyclo RGDyk (ExRg). Ex and ExRg rats received 8 weeks of treadmill running, and the rats in the ExRg group additionally were treated with a twice weekly injection of Cyclo RGDyk, an irisin receptor‐αV/β5 antagonist. At the end of the experiment, murine blood samples and heart tissues were collected and analyzed with methods of ELISA, Western blot, real‐time quantitative polymerase chain reaction, as well as immunofluorescence staining.

**Results:**

Exercise effectively mitigated T2DM‐related hyperglycemia, hyperinsulinemia, lipid dysmetabolism, and inflammation, which could be diminished by Cyclo RGDyk treatment. Additionally, exercise alleviated T2DM‐induced myocardial injury and excessive mitochondrial fission, whereas the beneficial effects were blocked by the administration of Cyclo RGDyk. T2DM significantly decreased serum irisin concentrations and fibronectin type III domain‐containing protein 5 (FNDC5)/irisin gene and protein expression levels in the rat heart, whereas exercise could rescue T2DM‐reduced FNDC5/irisin expression. Blocking irisin receptor signaling diminished the exercise‐alleviated mitochondrial fission protein expression and elevated AMPK phosphorylation.

**Conclusion:**

Exercise is effective in mitigating diabetes‐related insulin resistance, metabolic dysfunction, and inflammation. Irisin signaling engages in exercise‐associated beneficial effects on myocardial injury and excessive mitochondrial fission in diabetes rats involving elevated AMPK phosphorylation.

## Graphical abstract

Highlights

- Exercise reduced cardiac fibrosis and injuries, and improved over‐mitochondrial fission in type 2 diabetes mellitus (T2DM) rat hearts.
- Exercise increased serum irisin levels and cardiac irisin expression in T2DM rats; blocking irisin signaling by agent‐cyclo RGDyk diminished the protective effects of exercise on T2DM rat hearts.
- The ameliorative effect of exercise on myocardial injury in T2DM may be achieved through the regulation of the AMP‐activated protein kinase‐Drp1 signal by the myokine irisin to improve mitochondrial overfission.

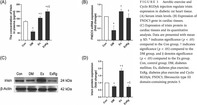

## 1 INTRODUCTION

Diabetes mellitus (DM) is a chronic metabolic disorder characterized by hyperglycemia, insulin resistance, and metabolic dysfunction.^1^ DM affects more than 463 million people worldwide in 2019, and this figure is expected to rise to 693 million by 2045.^2^, ^3^, ^4^ DM can directly cause structural and functional damage to the myocardium, such as myocardial inflammation, fibrosis, hypertrophy, and impaired contractility, which leads to a condition frequently referred to as diabetic cardiomyopathy (DCM).^5^ DCM, a specific form of cardiomyopathy, is more common and severe in type 2 DM (T2DM) cases and presents as a major contributor to the development and progression of heart failure and death in T2DM patients.^6^, ^7^ Given that DCM is associated with poor prognosis and high mortality, understanding its pathophysiology and molecular mechanisms is critical for the prevention and treatment of DCM.

Mitochondria are vital organelles for energy generation, particularly important for the heart because approximately 40% of the volume of cardiomyocytes is made up of mitochondria.^8^ Therefore, it is very important to maintain mitochondrial homeostasis, and any disturbance in the balance between mitochondrial fusion and fission will ultimately affect cardiac function.^9^ Mitochondria homeostasis is under the regulation of diverse proteins, such as dynamin‐related protein 1 (Drp1), a key protein that can migrate from the cytoplasm to the mitochondrial surface and bind to fission‐1 protein (Fis1) or mitochondrial fission factor (MFF) to cause mitochondrial fission.^10^ Excessive mitochondrial fission frequently leads to damage and dysfunction of mitochondria that subsequently trigger cell death.^11^ Hyperglycemia is a detrimental factor in the occurrence of excessive mitochondrial fission. It is reported that high glucose levels have been shown to increase mitochondrial fragmentation in divergent heart cell cultures,^11^, ^12^ and mitochondrial fission was associated with reduced heart function in patients with T2DM.^13^ Inversely, mitigating excessive mitochondrial fission has proven effective in preventing the worsening of diabetes‐related cardiomyopathy,^14^, ^15^, ^16^ providing a possible therapeutic strategy for DCM.

The benefits of exercise for people with T2DM and its associated cardiac damage are well established.^17^, ^18^ Growing evidence has revealed that regular exercise preserves heart function by preventing excessive mitochondrial fission of cardiomyocytes and maintaining mitochondrial dynamics.^19^, ^20^ Veeranki et al^21^ reported that 6 weeks of treadmill training restored mitochondrial homeostasis by reducing excessive mitochondrial fission in cardiomyocytes and protecting mitochondrial membrane integrity, thus enhancing myocardia contraction and reducing the structural and functional impairment of the diabetic murine heart. However, the relevant mechanism regulating the mitigation of exercise on mitochondrial fragments remains to be addressed.

During exercise, mitochondrial homeostasis is regulated by a range of signaling molecules, such as irisin and AMP‐activated protein kinase (AMPK). Irisin is a novel myokine, stimulated by exercise and derived from the cleavage of fibronectin type III domain‐containing protein 5 (FNDC5), which is abundantly expressed in the heart.^22^ Irisin has protective effects against T2DM‐associated cardiovascular disease by modulating mitochondrial dynamics and function.^23^ Chen et al^24^ revealed that intraperitoneal injection of recombinant (r)‐irisin or overexpression of FNDC5 in T2DM mice reduced mitochondrial fission and caspase‐dependent cardiomyocyte death, subsequently alleviating cardiac hypertrophy and fibrosis. AMPK is a key enzyme that regulates energy metabolism and mitochondrial function in organs and tissues,^11^ and it frequently interacts with irisin to regulate energy production. In in vitro cell culture experiments using human or mouse cells, irisin can activate the expression and phosphorylation of AMPK in response to chronic inflammatory stimuli such as hyperglycemia,^25^ lipopolysaccharide,^26^ among others,^27^, ^28^ but the protective mechanisms involved are unclear. Given the close relationship between irisin and AMPK in regulating mitochondrial dynamics and both regulated by the exercise,^29^ we speculated that irisin, by interacting with AMPK, plays a pivotal role in mediating the beneficial effects of exercise on excessive mitochondrial fission. Therefore, the purposes of this study were to (1) examine whether irisin is involved in the protective effect of exercise on DCM; and (2) if so, explore whether this protective effect is involved in activating AMPK signaling to attenuate Drp1‐relevant excessive mitochondrial fission.

## 2 MATERIALS AND METHODS

### 2.1 Animals and treatment

Forty 8‐week‐old male Wistar rats were purchased from the experimental animal center of Yangzhou University and were kept separately in special pathogen‐free conditions with a temperature of 24 ± 2°C, a relative humidity of 60 ± 10%, and a 12‐h light/dark cycle. All animal treatments are performed in compliance with regulations for the protection and use of laboratory animals that have been approved by Yangzhou University (No. 202303139). After adaptive feeding for 1 week, rats were randomly assigned into four groups: (1) control group (Con, *N* = 7); (2) DM group (DM, *N* = 7); (3) diabetes plus exercise group (Ex, *N* = 7); (4) diabetes plus exercise and Cyclo RGDyk (irisin receptor‐αVβ5 antagonist) (ExRg, *N* = 7). The body weight and fasting blood glucose (FBG) levels were determined weekly. After 12 h of fasting following the final exercise training, all rats were anesthetized with urethane and euthanized to collect samples.

### 2.2 Animal models

To establish T2DM rat models, the proportion of high‐fat feed was gradually added and then followed by high‐fat diet feeding (68% regular feed, 20% sucrose, 10% lard, 1.5% cholesterol, and 0.5% bile salts). After 4 weeks of the high‐fat diet, the rats were fasted (no water abstinence) for 12 h and intraperitoneally injected with a single dose of streptozotocin (STZ, 30 mg/kg), with the Con group rats injected with the same dose of sodium citrate buffer. The FBG level of rats was measured on the third and seventh days after injection, and if it was greater than 16.7 mmol/L, the modeling was successful.^30^

### 2.3 Drug injection protocol

One week after diabetic modeling, rats in the ExRg group received a tail intravenous injection of Cyclo RGDyk (GLPBIO Biological Company, USA) at a dosage of 2.5 mg/kg twice a week for 8 weeks, whereas the other groups were injected with the same amount of 10% dimethylsulfoxide solution.^31^

### 2.4 Exercise training protocol

For the aerobic exercise treadmill training, the rats underwent 8 weeks (5 days/week) of treadmill training at 5% inclination. Briefly, in the first week of the acclimatization exercise, the training time was raised gradually from 10 to 45 min, and the speed was progressively increased from 10 to 18 m/min. Following that, during the second to eighth weeks, treadmill training protocol was carried out at a fixed speed of 18 m/min for 45 min per day.

### 2.5 Biochemical analysis

Blood samples were collected from the abdominal artery of rats. Three blood cardiac enzymes, aspartate transaminase (AST), lactate dehydrogenase (LDH), and creatine kinase (CK) (myocardial damage indexes),^32^ as well as several indicators of lipid metabolism including triglyceride (TG), total cholesterol (TC), high‐density lipoprotein (HDL), and low‐density lipoprotein (LDL) levels, were measured by drawing blood in the fasting state and using the automatic biochemical analyzer (BK‐400, Biobase, China). Serum irisin (JL21442‐96 T, detection range: 15.6–1000 pg/mL, sensitivity: 7.6 pg/mL), fasting insulin (FINS) (JL10692‐96 T, detection range: 31.2–2000 pg/mL, sensitivity: 15.2 pg/mL), tumor necrosis factor‐α (TNF‐α) (JL13202‐96 T, detection range: 3.12–200 pg/mL, sensitivity: 1.75 pg/mL), and interleukin‐1β (IL‐1β) (JL20884‐96 T, detection range: 3.12–200 pg/mL, sensitivity: 1.51 pg/mL) levels were also assayed in accordance with the instructions provided in the monoclonal antibody‐based ELISA kits (Jianglaibio, China). Subsequently, the insulin resistance index (homeostatic model assessment for insulin resistance [HOMA‐IR]) of rats was calculated through FBG and FINS measurement data, which were obtained by using the formula HOMA‐IR = FBG (mmol/L) * FINS (mU/L)/22.5.

### 2.6 Histological staining

The 4% paraformaldehyde‐fixed heart tissues were dehydrated and transparent and then embedded in paraffin to obtain heart paraffin sections (5 μm). In these sections, hematoxylin and eosin staining (H&E) was used to identify specific structural alterations in hearts. In the meantime, Masson trichrome staining (G1346, Solarbio, China) and Sirius red staining (G1472, Solarbio, China) were performed to examine collagen deposition in hearts to visualize the degree of fibrosis. The staining outcomes were observed using a light microscope (Nikon, 400x magnification).

### 2.7 Immunofluorescence staining

Paraffin sections were exposed to antigen repair in an autoclave (pH = 8) loaded with EDTA repair solution, and endogenous peroxidase was eliminated using 3% hydrogen peroxide for 12 min to prevent false positives. After being blocked for 1 h with 5% skimmed milk, these sections were then incubated with primary antibody Drp1 (1:200, A2586, ABclonal) for 16 h at 4°C. The sections were then equilibrated to room temperature and then incubated with the fluorescent secondary antibody for 1 h, and hematoxylin and 4′,6‐diamidino‐2‐phenylindole were used to visualize the nuclei. The sections were observed and acquired using the two‐photon laser confocal fluorescence microscope (Carl Zeiss AG, Germany, 60X).

### 2.8 Western blot analysis

Heart tissues were lysed in the radio‐immunoprecipitation assay lysis mixture to get total protein, and the concentration of each sample was determined using the bicinchoninic acid assay protein analysis kit (P0011, Beyotime, China). The denatured proteins were then separated using 10% sodium dodecyl sulfate‐polyacrylamide gel electrophoresis and transferred to polyvinylidene fluoride (PVDF) membranes. After blocking PVDF membranes with 5% skimmed milk for 1 h at room temperature, transforming growth factor‐β1 (TGF‐β1; 1:500, sc‐130348, Santa Cruz, USA), irisin (1: 1500, SRP8039, Merck, China), AMPK (1:1000, AF6195, Beyotime, China), P‐AMPK (1: 2000, AF5908, Beyotime, China), Drp1 (1:2000, A2586, ABclonal, China), Fis1 (1:2000, CSB‐PA197834, Cusabio, China), MFF (1:2000, GB114102, Servicebio, China), and β‐actin (1:20000, 66 009‐1‐lg, Proteintech, China) were detected using the immunoblot at 4°C. The density of protein bands was evaluated with Image‐Pro Plus software (version 6.0, Media Cybernetics, USA) and normalized to the equivalent density of the housekeeping protein β‐actin.

### 2.9 Real‐time quantitative reverse transcription polymerase chain reaction (RT q‐PCR) analysis

Total mRNA from heart tissues was isolated using Trizol reagent and an equal amount of RNA was reverse transcribed into cDNA using Prime Script RT q‐PCR kit. For quantitative PCR, the reactants were added sequentially in a 96‐well optical reaction plate, covered with an optical membrane, and then centrifuged instantaneously at 2000 rpm so that the reactants mixed system was completely located at the bottom of the optical wells of the reaction plate. The reflector plate was placed in the PCR instrument and amplification was performed according to the prescribed procedure. After the procedure, the effective 2^−ΔΔCt^ values were analyzed and normalized to the transcript level of glyceraldehyde‐3‐phosphate dehydrogenase (GAPDH) as a control. All primers used are shown in Table 1.

**TABLE 1:** The sequences of primers used for real‐time quantitative reverse transcription polymerase chain reaction.

Gene	Primer	Sequence
*FNDC5*	Forward	5′‐AAGTGGTCATTGGCTTTGC‐3′
	Reverse	5′‐GTTATTGGGCTCGTGT‐3′
*Drp1*	Forward	5'‐CAGCGAGATTGTGAGGTTATTGA‐3′
	Reverse	5′‐CGGATTCAGTCAGAAGGTCATC‐3′
*Fis1*	Forward	5′‐ATCCGTAGAGGCATCGTG‐3′
	Reverse	5′‐CCTTGAGCCGGTAGTTG‐3′
*MFF*	Forward	5′‐CTGCCGCCACTTCTAATCCTCATC‐3′
	Reverse	5′‐TCAATGAAGCCGCATCTACCACAG‐3′
*GAPDH*	Forward	5′‐CAGTGCCAGCCTCGTCTCAT‐3′
	Reverse	5′‐AGGGGCATCCACAGTCTTC‐3′

### 2.10 Statistical analysis

The data collected from at least three replicate independent experiments were processed using the Graph Pad Prism (version 9.4.1, CA, USA), and the results are presented with mean value ± SD notation. For evaluating between‐group differences, one‐way analysis of variance was used for comparison with a Bonferroni post hoc test. *p* < .05 was considered to be a statistically significant difference.

## 3 RESULTS

### 3.1 Exercise increases body weight and decreases hyperlipemia, hyperglycemia, and insulin resistance in diabetic rats

Before inducing T2DM, there was no discernible difference in the baseline body weight and circulating levels of FBG between groups of rats. To establish T2DM models, rats were fed a high‐fat diet for 4 weeks and then injected intraperitoneally with a single dose of STZ. The body weight of rats was not significantly different between the four groups after inducing T2DM. However, FBG levels increased markedly in diabetic groups compared to the control group meeting the criteria for inducing T2DM (Table 2).^33^ After 8 weeks of intervention, T2DM rats had obviously lower body weight, lower serum HDL‐C levels, and higher TC, TG, and LDL‐C levels compared with the control rats, implying obvious abnormalities in lipid metabolism. Moreover, diabetic rats also exhibited remarkable hyperglycemia and hyperinsulinemia in contrast to littermate control rats, indicating the existence of insulin resistance. Furthermore, we measured serum markers of inflammation to evaluate the inflammatory status of the rats. Intriguingly, rats with T2DM exhibited markedly elevated concentrations of TNF‐α and IL‐1β compared to the control group. To evaluate the impact of exercise on diabetes‐associated metabolic dysfunction and inflammation, the T2DM rats underwent a training protocol that has been described previously. Apparently, the 8‐week exercise intervention was effective in ameliorating weight loss, hyperlipidemia, hyperglycemia, insulin resistance, and inflammation in T2DM rats. However, the exercise‐relevant beneficial effects could be reversed by the administration of Cyclo RGDyk, a blocking agent targeting the irisin receptor‐αV/β5 pathway.

**TABLE 2:** Weights and serum indexes of rats between groups.

Indexes	Con	DM	Ex	ExRg
Weight__base_ (g)	266.5 ± 5.4	273.1 ± 6.4	270.3 ± 9.1	270.9 ± 9.6
Weight__pre_ (g)	339 ± 10.06	336.8 ± 14.94	349.3 ± 15.36	343.3 ± 11.67
Weight__post_ (g)	437 ± 21.3	323.8 ± 27.6*	370.4 ± 25.7* ^,^ ^†^	354.3 ± 29*
TC (mmol/L)	1.94 ± 0.06	3.99 ± 0.16*	2.05 ± 0.13^†^	3.67 ± 0.12* ^,^ ^†^ ^,^ ^§^
TG (mmol/L)	1.1 ± 0.18	3.2 ± 0.23*	1.41 ± 0.26^†^	2.86 ± 0.13* ^,^ ^†^ ^,^ ^§^
LDL‐C (mmol/L)	0.7 ± 0.02	1.3 ± 0.09*	0.64 ± 0.09^†^	0.79 ± 0.08^†^ ^,^ ^§^
HDL‐C (mmol/L)	0.68 ± 0.03	0.33 ± 0.08*	0.8 ± 0.1* ^,^ ^†^	0.41 ± 0.04* ^,^ ^†^ ^,^ ^§^
FBG__pre_ (mmol/L)	5.61 ± 0.28	20.8 ± 1.69*	20.11 ± 1.9*	20.41 ± 1.26*
FBG__post_ (mmol/L)	5.68 ± 0.4	21.9 ± 1.83*	17.67 ± 2.57* ^,^ ^†^	19.12 ± 1.96*
FINS (mU/L)	5.58 ± 0.62	15.4 ± 1.85*	8.64 ± 1.23* ^,^ ^†^	12.28 ± 1.44* ^,^ ^†^ ^,^ ^§^
HOMA‐IR	1.45 ± 0.31	14.73 ± 1.33*	7.42 ± 1.25* ^,^ ^†^	11.6 ± 1* ^,^ ^†^ ^,^ ^§^
TNF‐α (pg/mL)	77.26 ± 8.47	148.3 ± 12.75*	112.8 ± 12.46* ^,^ ^†^	130.8 ± 9.86* ^,^ ^§^
IL‐1β (pg/mL)	65.48 ± 5.25	99.08 ± 10.54*	80.24 ± 5.95* ^,^ ^†^	91.72 ± 3.13* ^,^ ^§^

*Note*: Data are presented with mean ± SD.

Abbreviations: Con, control group; DM, diabetes mellitus; Ex, diabetes plus exercise; ExRg, diabetes plus exercise and Cyclo RGDyk; FBG, fasting blood glucose; FINS, fasting insulin; HDL‐C, high‐density lipoprotein cholesterol; HOMA‐IR, homeostasis model assessment‐insulin resistance; IL‐1β, interleukin‐1β; LDL‐C, low‐density lipoprotein cholesterol; TC, total cholesterol; TG, triglyceride; TNF‐α, tumor necrosis factor‐α.

* Presents significant (*p* value <.05) compared to Con group.

^†^ Indicates significant (*p* value <.05) compared to DM group.

^§^ Denotes significant (*p* value <.05) compared to Ex group.

### 3.2 Exercise improves diabetes‐related myocardial injury but diminished by cyclo RGDyk treatment

**FIGURE 1:**
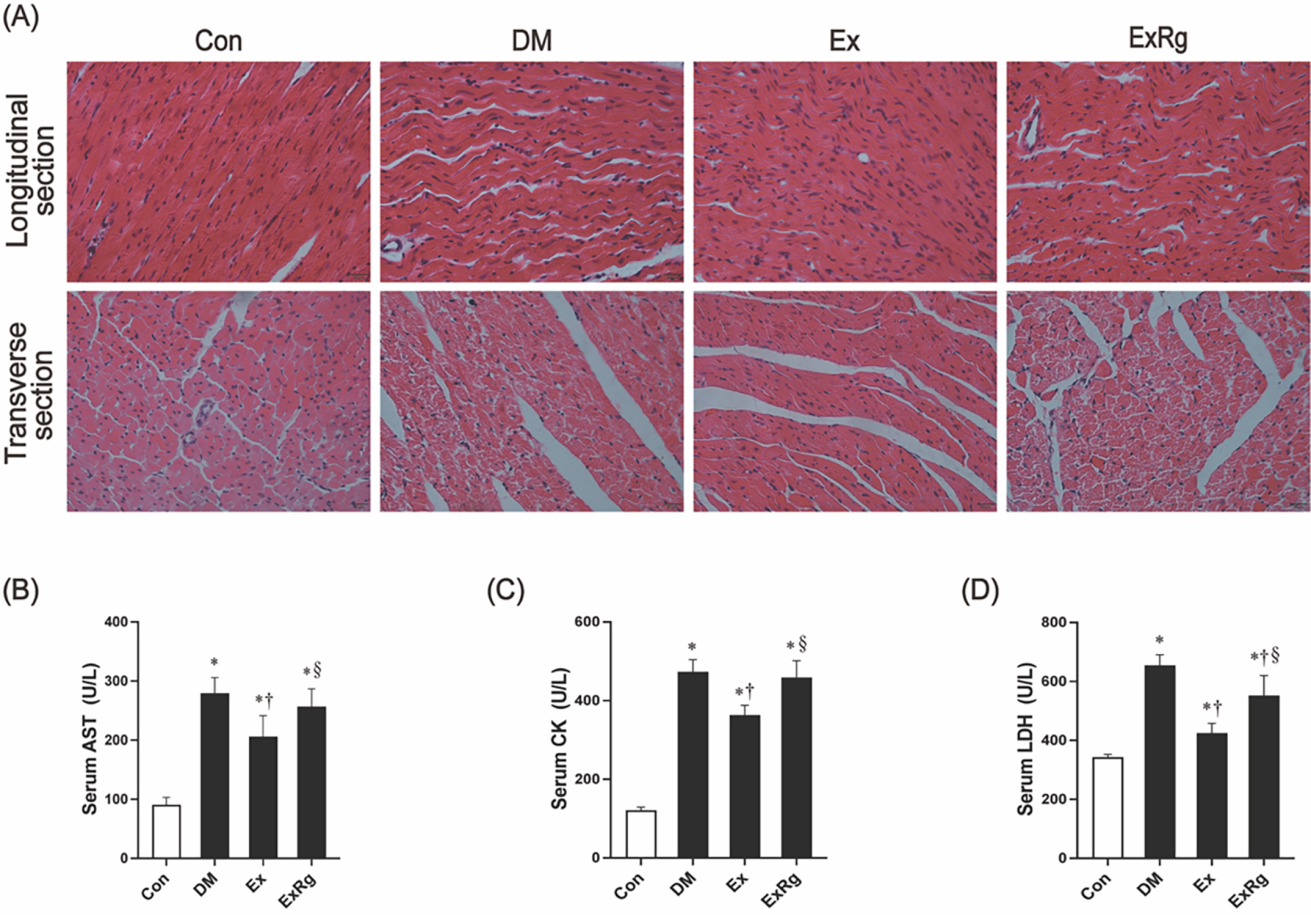
The effect of exercise on diabetic heart damage. (A) Longitudinal and transverse H&E staining images of rats (200X). (B, C, D) Serum levels of LDH, CK, and AST, markers of myocardial injury. Data are presented with mean ± SD. * indicates significance (*p* < .05) compared to the Con group, † indicates significance (*p* < .05) compared to the DM group, and § denotes significance (*p* < .05) compared to the Ex group. AST, aspartate aminotransferase; CK, creatine kinase; Con, control group; DM, diabetes mellitus; Ex, diabetes plus exercise; ExRg, diabetes plus exercise and Cyclo RGDyk; H&E, hematoxylin and eosin staining; LDH, lactate dehydrogenase.

Myocardial morphology was analyzed to determine the structural change in the rat heart in the appearance of T2DM, as well as its response to the exercise intervention. As shown by H&E staining (Figure 1A), the cardiomyocytes were orderly aligned with regular shape and size in control rats. In contrast, T2DM rats showed disorganized myocardial fibers and deformed, fragmented myocardial cells, accompanied by infiltration of inflammatory cells and an enlarged space of extracellular interstitium in tissues. Eight‐week exercise training restored the damaged myocardial morphology and structure of T2DM rats. Previous studies have reported that irisin plays a pivotal role in myocardial function and repairment^34^, ^35^ and its secretion is mediated by exercise.^36^, ^37^ We, therefore, suspected that the exercise‐related beneficial effects on the diabetic rat heart are under the regulation of irisin signaling. Accordingly, when exercised rats received twice‐weekly injections of Cyclo RGDyk for 8 weeks, the exercise‐associated favorable effects were diminished. Meanwhile, we also detected elevated circulating levels of myocardial enzymes, including AST, CK, and LDH, in T2DM rats (Figure 1B–D). Exercise intervention significantly ameliorated the enhanced myocardial enzymes in diabetic rats, but the beneficial changes were diminished by Cyclo RGDyk administration. Taken together, these data indicated a potential role of irisin signaling in mediating the impact of exercise on diabetic heart damage.

### 3.3 Exercise alleviates diabetes‐induced myocardial fibrosis via irisin receptor signaling

**FIGURE 2:**
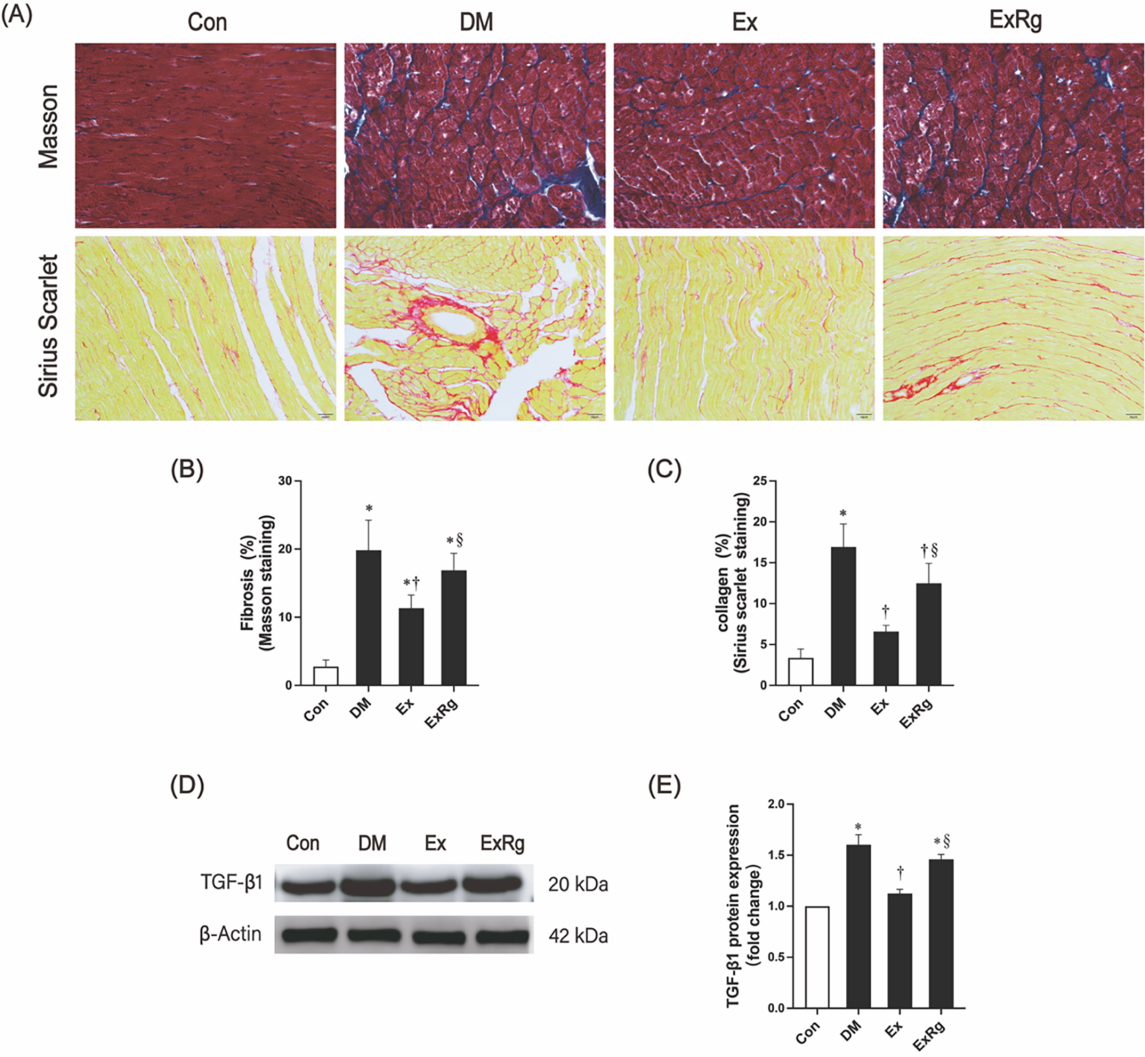
The effects of exercise on myocardial fibrosis. (A) Representative images of Masson trichrome staining and Sirius red staining (200X). (B) Masson trichrome staining results were quantified by determining the percentage of the blue region to the overall area of the picture. (C) Calculate the percentage of the red region to the entire picture area to quantify the outcomes of the Sirius red staining. (D, E) Expression of TGF‐β1 protein and the quantitative result. Data are presented with mean ± SD. * indicates significance (*p* < .05) compared to the Con group, † presents significance (*p* < .05) compared to the DM group, and § denotes significance (*p* < .05) compared to the Ex group. Con, control group; DM, diabetes mellitus; Ex, diabetes plus exercise; ExRg, diabetes plus exercise and Cyclo RGDyk; TGF‐β1, transforming growth factor‐β1.

Myocardial fibrosis is a featured pathology of DCM.^38^ Our study employed Masson trichrome staining and Sirius red staining (Figure 2A–C) to assess the extent of fibrosis in rat hearts. In contrast to the control rats, T2DM rats showed an increment in fibrosis (Masson trichrome staining) and collagen deposition area (Sirius red staining) in heart tissue, suggesting that the occurrence of T2DM could induce serious fibrosis in the myocardium. Eight‐week aerobic exercise training significantly ameliorated myocardial fibrosis. The exercise‐related improvement, however, diminished with the 8‐week Cyclo RGDyk treatment. Previous studies demonstrated that TGF‐β1, a crucial protein regulating the synthesis and degradation of interstitial collagen fibers, was abundantly expressed in the fibrotic heart.^39^ According to Western blot analysis (Figure 2D, E), the diabetic rat heart showed considerably higher TGF‐β1 protein expression levels than the control rat heart. Exercise intervention downregulated TGF‐β1 expression levels, which subsequently were elevated by an 8‐week Cyclo RGDyk intervention. Collectively, our findings demonstrate that irisin receptor signaling may be involved in exercise‐induced improvement in myocardial fibrosis in diabetic rats.

### 3.4 The myocardial expression of FNDC5/irisin is regulated by exercise and cyclo RGDyk treatment

**FIGURE 3:**
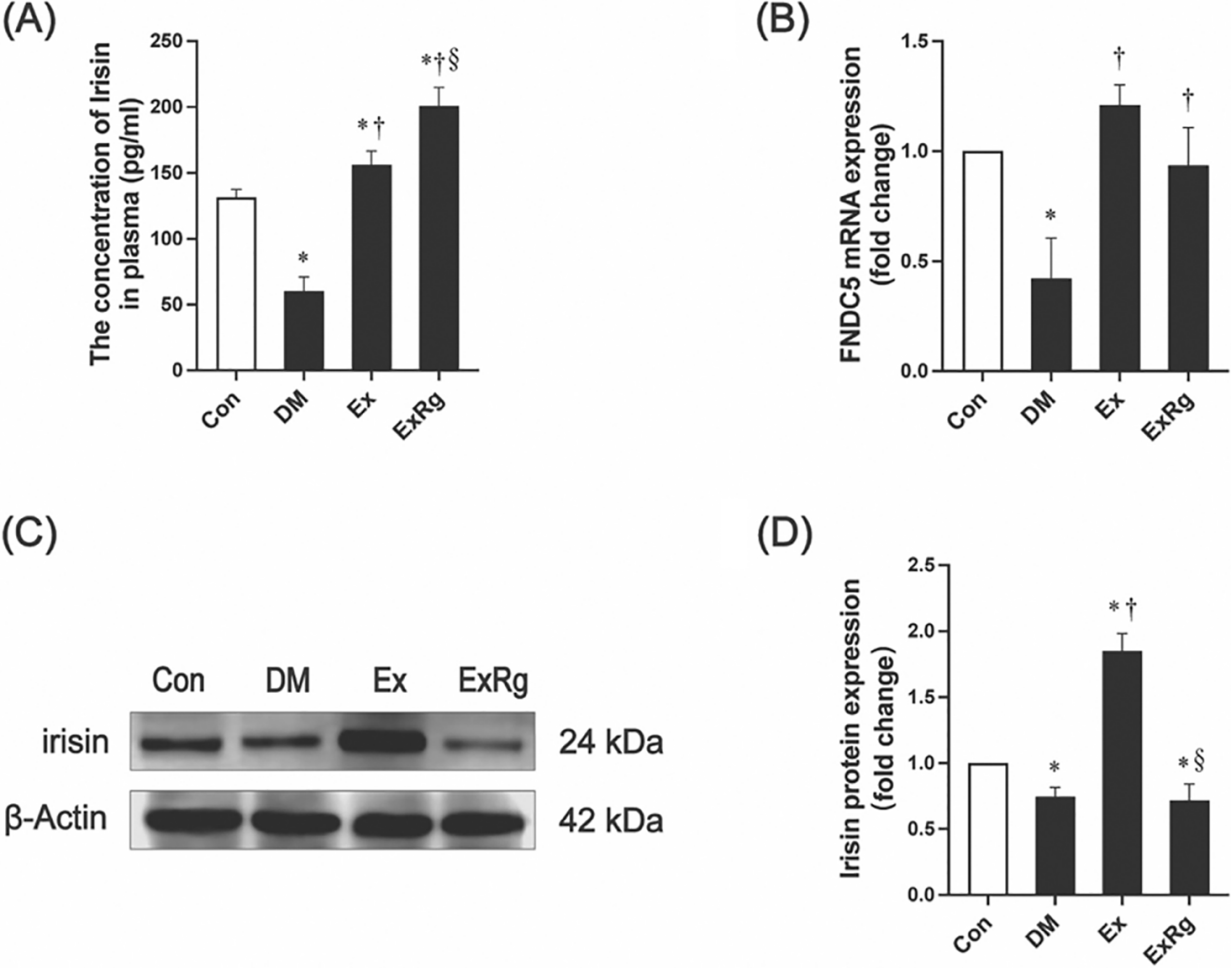
Aerobic exercise and Cyclo RGDyk injection regulate irisin expression in diabetic rat heart tissue. (A) Serum irisin levels. (B) Expression of FNDC5 gene in cardiac tissues. (C) Expression of irisin protein in cardiac tissues and its quantitative analysis. Data are presented with mean ± SD. * indicates significance (*p* < .05) compared to the Con group, † indicates significance (*p* < .05) compared to the DM group, and § denotes significance (*p* < .05) compared to the Ex group. Con, control group; DM, diabetes mellitus; Ex, diabetes plus exercise; ExRg, diabetes plus exercise and Cyclo RGDyk; FNDC5, fibronectin type III domain‐containing protein 5.

First, we analyzed the serum irisin levels in diabetic rats (Figure 3A). Our findings revealed a dramatic decrease in the serum irisin levels of rats in the DM group compared to those in the Con group. After 8‐week exercise training, serum irisin levels in T2DM rats considerably increased. The administration of Cyclo RGDyk to exercising diabetic rats elevated serum irisin levels, which is in line with a previous report.^27^ As irisin is a tissue‐specific expression protein, we determined the expression level of irisin in cardiac tissue to examine its potential role in exercise‐related cardioprotection. RT q‐PCR (Figure 3B) and Western blot analyses (Figure 3C,D) revealed a significantly decreased expression of FNDC5 mRNA and irisin protein in T2DM rat heart tissue compared to the Con group. Exercise training markedly upregulated the FNDC5 mRNA and irisin protein expression, but the treatment of synthetic RGDyk peptide decreased irisin protein expressions, without obvious effect on FNDC5 mRNA levels. These results suggest that the appearance of T2DM decreased myocardial expression of irisin that could be restored by exercise intervention. Given the essential role of irisin in regulating cardiac function,^34^, ^35^, ^37^ irisin may be involved in the exercise‐relevant beneficial effects on diabetic rat heart.

### 3.5 Exercise mitigates excessive mitochondrial fission in diabetic rat hearts via the irisin/AMPK signaling pathway

**FIGURE 4:**
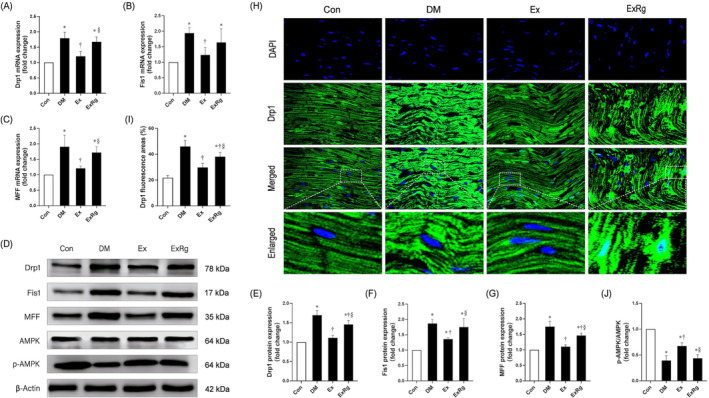
Irisin mediates the effect of exercise on mitochondrial fission in diabetic rat hearts involving the phosphorylation of AMPK. (A, B, C) mRNA levels of Drp1, Fis1, and MFF in rat hearts. (D, E, F, G) Protein expression levels of Drp1, Fis1, and MFF in rat hearts and their quantitative analysis. (H, I) Immunofluorescence staining of Drp1 and its quantitative analysis (1000X). (J) Quantitative analysis of AMPK phosphorylation levels in the heart. Data are presented with mean ± SD. * indicates significance (*p* < .05) compared to the Con group, † presents significance (*p* < .05) compared to the DM group, and § denotes significance (*p* < .05) compared to the Ex group. AMPK, AMP‐activated protein kinase; Con, control group; Drp1, dynamin‐related protein 1; Ex, diabetes plus exercise; ExRg, diabetes plus exercise and Cyclo RGDyk; Fis1, fission‐1 protein; MFF, mitochondrial fission factor.

Because excessive mitochondrial fission is a hallmark of diabetic pathological changes and is a detrimental factor for myocardial pathology,^40^ we hypothesized that exercise improves cardiac function possibly via the mitigation of elevated mitochondrial fission. The mRNA (Figure 4A–C) and protein levels (Figure 4D–G) of Drp1, Fis1, and MFF were significantly increased in T2DM rat hearts compared to the control group, whereas the diabetes‐enhanced mitochondrial fission proteins were reversed by exercise training. We further blocked irisin receptor signaling by administration of Cyclo RGDyk. The results revealed that Drp1, Fis1, and MFF mRNA and protein expression remarkably increased again, indicating the involvement of irisin receptor signaling in exercise‐induced beneficial effects. Additionally, confocal microscopy analysis demonstrated a considerable elevation of Drp1 fluorescence intensity in cardiomyocytes of T2DM rats compared to the control, indicating the existence of excessive fission of mitochondria in rat heart tissue (Figure 4H,I). Drp1 expression was considerably decreased in the Ex group compared with the DM group. The hearts of ExRg group rats treated with Cyclo RGDyk showed a significant increase in the fluorescence intensity of Drp1 in contrast to those of Ex group rats. According to these results, reducing excessive mitochondrial fission is a key mechanism for exercise to ameliorate DCM, and irisin signaling may play a crucial role in regulating the therapeutic effects of exercise. Given the close relationship between AMPK signaling and irisin in the mediation of mitochondrial function,^29^ we further determined whether the improvement of mitochondrial fission induced by exercise is involved in the activation of AMPK and regulated by irisin signaling. In T2DM rats, phosphorylated AMPK expression was depleted in contrast to the Con group, whereas exercise training intervention boosted p‐AMPK expression (Figure 4D,J). However, Cyclo RGDyk injection decreased the expression levels of exercise‐elevated p‐AMPK protein. The results proposed a possible role of irisin signaling involved in exercise‐ameliorated excessive mitochondrial fission in diabetic rat myocardium through upregulating AMPK phosphorylation.

## 4 DISCUSSION

Our findings demonstrated that exercise can reduce heart fibrosis and improve DCM in T2DM rats. This study also reveals that exercise may activate irisin, which in turn triggers AMPK phosphorylation. This process can prevent Drp1 from causing excessive mitochondrial fission in the cardiac tissue of T2DM rats.

The positive effects of regular exercise on cardiovascular health are well established.^41^ Shawahna et al^17^ have shown that exercise training is an effective strategy for preventing or alleviating T2DM and its related cardiovascular complications. In accordance with those findings, our results revealed that exercise significantly reduced myocardial fibrosis and cardiac enzyme levels in T2DM rats, supporting the therapeutic implication of exercise for DCM.

As a novel myokine, irisin has attracted considerable attention for its function in health and diseases, especially metabolic disorders such as T2DM.^42^ Given the close relationship between irisin and physical activity, we assumed that the irisin‐mediated pathway might play a role in the influences of exercise in DCM. In agreement with this hypothesis, our study found that exercise improved cardiac outcomes and increased FNDC5/irisin levels in T2DM rat hearts and blocking αV/β5 with Cyclo RGDyk reduced the expression of markers related to mitochondrial fission in the heart tissue. As a receptor of irisin, αV/β5 has been detected in various organs and tissues, including cardiomyocytes.^24^, ^43^, ^44^, ^45^ Kim et al demonstrated that the αV/β5 effectively blocked the signal transduction induced by irisin.^43^ Therefore, it is indicated that irisin signaling probably is engaged in the beneficial effects of exercise on DCM. Formigari et al recently investigated the role of irisin in mediating the effects of exercise on renal function in diabetic rats. Their findings revealed that Cyclo RGDyk treatment induced a remarkable increment of serum irisin in diabetic rats and a mild elevation of irisin in diabetic rats with exercise.^27^ Our study also indicated that Cyclo RGDyk treatment elevated serum irisin levels in diabetic rats undergoing exercise intervention. However, the specific mechanism underlying this effect remains unclear. Considering that the data on the effects of Cyclo RGDyk treatment on serum irisin are currently lacking, they need to be investigated in future studies.

Nikolajczyk has suggested that T2DM is a condition of inflammation and that chronic inflammation impairs mitochondrial dynamics.^24^ Mitochondrial dynamics is the continuous process of merging and splitting of mitochondria for quality control. Recently, Wu and colleagues^46^ have reported that excessive mitochondrial splitting causes mitochondrial dysfunction, which is crucial for the development and progression of DCM.^46^ Accordingly, our study confirmed that, by alleviating excessive mitochondrial fission, the exercise intervention showed effectiveness in the improvement of T2DM and its associated cardiac damage.

AMPK acts as a crucial energy sensor for maintaining cellular and mitochondrial homeostasis. Its regulatory effects on cardiac function include modulating cardiac energy equilibrium and myocardial signal transduction^47^ and are strongly associated with mitochondrial fission.^48^, ^49^

AMPK activation leads to AKAP1 phosphorylation at Ser103, which enables its interaction with protein kinase A. This in turn results in Drp1 phosphorylation at Ser637, which reduces its affinity for mitochondrial receptors and prevents excessive mitochondrial fission.^50^ In line with the function of AMPK in mitochondrial fission, we observed a reduction in the expression of p‐AMPK protein and a remarkable augment of mitochondrial fission markers in T2DM rat hearts. Moreover, exercise training increased p‐AMPK levels and decreased Drp1‐mediated mitochondrial fission in the myocardium of T2DM rats, but these effects were abolished by blocking irisin signaling. Together, these results strongly support the notion that exercise can improve DCM by preventing excessive mitochondrial splitting, which involves irisin signaling and its link to AMPK activation.

In this paper, we have demonstrated that exercise can protect the heart from T2DM‐induced damage and mitochondrial dysfunction in rats and that this protective effect involves the activation of irisin signaling and AMPK phosphorylation. However, our research has some limitations that need to be addressed in future studies. First, the Cyclo RGDyk peptide that we used to block the irisin receptor αV/β5 is not strictly specific and may also interfere with the expression of another integrin, αV/β3, which could cause nonspecific effects. Second, our study mainly focused on in vivo experiments, which means that we did not examine the specific effects of irisin on different cell types of the heart such as cardiomyocytes, endothelial cells, and fibroblasts, nor did we measure fibrosis markers such as fibronectin and collagen in cardiac tissue. Furthermore, the absence of hemodynamic data, such as blood pressure, is also a limitation as previous studies have revealed the involvement of irisin in the positive impact of exercise on blood pressure in diabetic mice.^27^, ^51^, ^52^ Therefore, more studies are required to clarify these issues and to explore the potential of irisin as a novel therapeutic strategy for improving DCM.

## AUTHOR CONTRIBUTIONS

The study was designed and the first draft manuscript was written by Bin Wang and Renqing Zhao. Material preparation, data collection, and analysis were undertaken by Chen Zhao, Yuanxin Wang, Xin Tian, Junjie Lin, Baishu Zhu, Yalan Zhou, Xin Zhang, Nan LI, Yu Sun, andHaocheng Xu. The final manuscript was read and approved by all authors.

## FUNDING INFORMATION

Partial support for this study was provided by the Natural Science Foundation of Jiangsu Province (BK20201435).

## CONFLICT OF INTEREST STATEMENT

The authors state that the study was conducted without any commercial or financial affiliations that might indicate a potential conflict of interest.

## Data Availability

The authors of this article will make the raw data supporting their conclusions available, without any hesitation or reservation.
